# Light–Matter interactions on the nanoscale

**DOI:** 10.3762/bjnano.9.201

**Published:** 2018-08-10

**Authors:** Mohsen Rahmani, Chennupati Jagadish

**Affiliations:** 1 Research School of Physics and Engineering, The Australian National University, Canberra, ACT 2601, Australia

**Keywords:** light-matter interactions, nano-optics, nanophotonics, plasmonics

At the beginning of the 20th century, researchers began harnessing the capabilities of electricity and magnetism. Today we are standing in a similar position as we contemplate the emergence of light–matter interactions at the nanoscale and how it will continue its exponential growth over the coming years. This research field, also called nanophotonics or nano-optics, is a subject of rapidly increasing scientific importance: controlling light–matter interactions beyond the diffraction limit. In contrast to optics, which is the concept of light rays, including absorption, transmission and reflection, photonics is the concept of light science, including emission, detection, amplification and control of light. Photonics offers a wide range of applications in different areas ranging from sensing and imaging, to solar cells and optical communication [1]. It is no surprise that many consider photonics to be the technology revolution of the 21st century.

Laser processing of thin-film multilayer structures has been one of the initial research directions in photonics [2]. This technique has been employed for many applications, including but not limited to the fabrication of polycrystalline silicon (poly-Si) thin-film transistors or MEMS/NEMS devices, as well as biomedical engineering [3–4]. Subsequently, with advances in photolithography, the microscale structure of materials began to attract much research interest due to their unique capability to interact with an applied electromagnetic wave in the radio frequency and terahertz regions [5]. This part of the spectrum has unique properties, such as being non-ionizing and subject to considerably less Rayleigh scattering [6]. In the last decade, by exploiting the rapid progress in computational and characterisation techniques, together with advances in techniques for the fabrication nanostructures, researchers performed detailed studies on the light–matter interaction in the visible and near-infrared region for nanostructures with dimensions on the order of (or even smaller than) the wavelength [7].

Metallic nanoparticles have been most heavily studied particles to bridge the gap between conventional optics and highly integrated nanophotonic components via stimulating the oscillation of free electrons on the surface, so-called surface plasmons [8]. Stimulated by the flourishing field of plasmonics, many novel effects have been suggested and even demonstrated, including super-scattering, clocking, control of the scattering direction, artificial antiferromagnetism, etc. [9]. Meanwhile, inverse plasmonics, that is, apertures in metallic films, has also been the subject of ongoing research [10]. Although many plasmonic applications have faced fundamental limitations (due to the ohmic losses in metals), the knowledge is still valuable for developing new strategies for light–matter interactions on the nanoscale.

High refractive index dielectric [11] and semiconductor [12] nanostructures have been recently exploited as an alternative to plasmonics [13]. Dielectrics and semiconductors benefit from negligible resistive losses. This advantage allows excitation at large light intensities for significant concentration of light at the nanoscale, which is not limited to interfaces. Such nanostructures have multipolar characteristics of both electric and magnetic resonant modes that could aid in the engineering of light behaviour at the nanoscale. Among various applications of dielectric nanostructures, metasurfaces, composed of a single or a few stacked layers of subwavelength nanostructures/particles, are growing in popularity [14]. This is because metasurfaces can offer a diverse range of applications, including sensing and optical tuning, dispersion engineering and polarization manipulation [14].

Recently, it has been demonstrated that light–matter interactions at the nanoscale can even be induced via sub-nanometer materials [15–16], for example, graphene [17]. The interaction of graphene with electromagnetic radiation is fascinating due to the two-dimensional confinement of electrons and the exceptional band structure of graphene. Graphene has a simple band structure with zero band gap, but its optical response is nontrivial. Subsequently, other two-dimensional (2D) materials, such as transition-metal dichalcogenides (TMDCs) or hexagonal boron nitride (hBN) [10] have also emerged as interesting platforms for nanophotonics. TMDCs, with their intrinsically broken inversion symmetry in crystal structure, have shown many advanced optical properties with potential applications such as in valleytronics. On the other hand, hBN has promising hyperbolic properties as well as the ability to host a range of single photon emitters (SPEs) for quantum photonic applications.

In summary, the field of photonics is ever growing and the life of people will be greatly influenced by the developments in this area. This Thematic Series can guide readers in understanding the physics of light matter–interaction with various kinds of nanostructures, including metallic (plasmonic), dielectric and semiconductor, 2D, as well as hybrid nanostructures [18]. Meanwhile, readers can become more familiar with the cutting edge advances in this respect.

Mohsen Rahmani and Chennupati Jagadish

Canberra, July 2018
